# Solute Carrier Family 27 Member 4 (SLC27A4) Enhances Cell Growth, Migration, and Invasion in Breast Cancer Cells

**DOI:** 10.3390/ijms19113434

**Published:** 2018-11-01

**Authors:** Meng-Chi Yen, Shih-Kai Chou, Jung-Yu Kan, Po-Lin Kuo, Ming-Feng Hou, Ya-Ling Hsu

**Affiliations:** 1 Department of Emergency Medicine, Kaohsiung Medical University Hospital, Kaohsiung Medical University, Kaohsiung 807, Taiwan; yohoco@gmail.com; 2 Graduate Institute of Clinical Medicine, College of Medicine, Kaohsiung Medical University, Kaohsiung 807, Taiwan; kuopolin@seed.net.tw; 3 Graduate Institute of Medicine, College of Medicine, Kaohsiung Medical University, Kaohsiung 807, Taiwan; q2982267@gmail.com; 4 Department of Breast Surgery, Kaohsiung Medical University Hospital, Kaohsiung Medical University, Kaohsiung 807, Taiwan; kan890043@gmail.com

**Keywords:** solute carrier family 27 member 4 (SLC27A4), fatty acid transport protein 4 (FATP4), very long-chain acyl-CoA synthetases member 4 (ACSVL4), breast cancer, fatty acid transporter, proliferation, migration, invasion, lipid metabolism

## Abstract

Fatty acid metabolism is important in the regulation of breast cancer progression. Some of the proteins involved in fatty acid transport have been demonstrated to promote the proliferation, migration, and invasion in breast cancer cells. Solute carrier family 27 member 4 (SLC27A4) is a fatty acid transporter protein and is related to very long chain acyl-CoA synthetase activity. In the present study, bioinformatic analysis revealed that relatively high SLC27A4 expression was observed in all subtypes of breast tumor tissues when compared to normal breast tissues. Silencing SLC27A4 expression significantly reduced uptake of free fatty acids in two breast cancer cell lines, Hs578T and MDA-MB-231. Cell growth inhibition was observed in SLC27A4-silenced Hs578T and cell cycle was arrested at G2/M. In addition, the capacity of migration and invasion decreased in both cell lines after knockdown of SLC27A4. The epithelial–mesenchymal transition signaling pathway was inhibited because protein expression of Slug, vimentin, α-smooth muscle actin, and other regulators was lower than that in control cells. Taken together, our results confirm that high SLC27A4 is associated with tumor progression in breast cancer cells. It is worth investigating whether SLC27A4 serves a diagnostic marker and therapeutic target in further studies.

## 1. Introduction

Among all types of women’s cancers, breast cancer has the most new cases of diagnosed cancer type and is the second cause of cancer-related mortality worldwide [1]. Currently, dysregulation of metabolic pathways, including fatty acid metabolic pathways, is considered as a risk factor for promoting breast cancer progression [2]. Fatty acid metabolism comprises multiple pathways including fatty acid transport, de novo synthesis, fatty acid oxidation, etc., and emerging evidence has indicated that some of the fatty acid metabolic enzymes are related to different subtypes of breast cancer [3]. Fatty acid binding proteins (FABP) are a family of proteins that bind long-chain fatty acids and are involved in facilitating transport and uptake of lipids. Overexpression of FABP5 and FABP7 is associated with triple-negative breast cancer and basal-like breast cancer [4,5,6]. Acyl-CoA synthetase long-chain family member 4 (ACSL4) activity leads to long-chain fatty acid transport and long-chain fatty-acid-coenzyme A ligase. High ACSL4 expression is inversely associated with estrogen receptor expression and high ACSL4 expression is a biomarker for an aggressive breast cancer phenotype [7,8,9]. Thus, these enzymes might serve as therapeutic targets and diagnostic markers in different subtypes of breast cancer.

CD36 is a transmembrane protein and mediates fatty acid uptake. Recent studies indicate that high CD36 expression is detected in breast cancer and CD36 function is important for cell growth in breast cancer cells and metastasis in metastasis-initiating breast cancer cells [10,11]. Therefore, the fatty acid transport pathway is important for breast cancer progression. Except for CD36, ACSL, and FABP, solute carrier family 27 (SLC27) is also involved in the process of long-chain fatty acid uptake. SLC27, also named fatty acid transport proteins (FATP) or very long-chain acyl-CoA synthetases (ACSVL), is a family of six members (SLC27A1 through SLC27A6) for uptake of long-chain fatty acids [12]. Each protein has different specific preferred substrates and tissue distribution [13]. In addition, SLC27 family proteins, especially SLC27A1 (FATP1) and SLC27A4 (FATP4), display acyl-CoA synthetase (ACS) activity, which links to fatty acid synthesis, β-oxidation, and phospholipid synthesis [12,14]. This suggests that SLC27 family proteins are involved in regulation of fatty acid uptake and down-streaming of lipid metabolic processes.

Currently, the role of SLC27 family proteins is not fully understood in breast cancer although the role of ACSL, FABP, and CD36 has been investigated. The aim of this study is to investigate whether SLC27 family proteins are associated with progression of breast cancer, including cell growth, migration, invasion, and potential regulatory mechanism in breast cancer cells.

## 2. Results

### 2.1. Relatively High SLC27A4 Expression Was Detected in Breast Cancer Tissue

**Figure 1:**
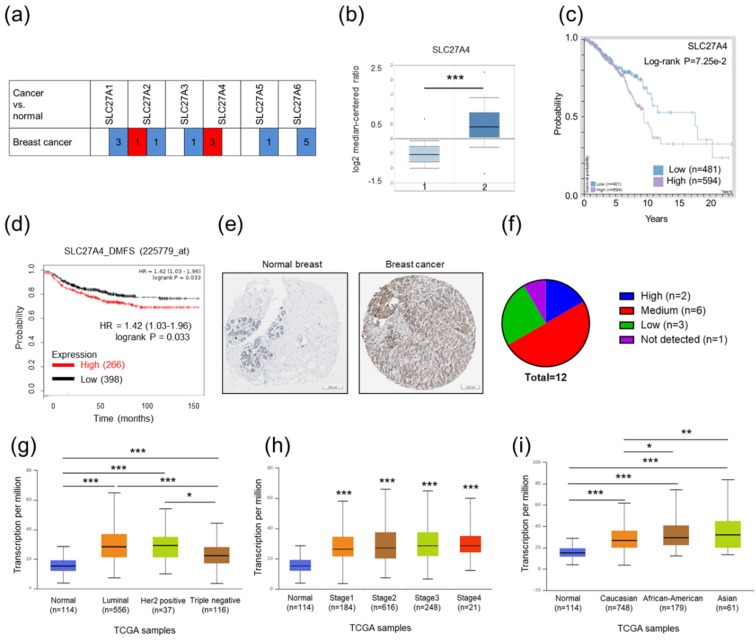
SLC27A4 expression in breast cancer and noncancer tissues. (**a**) SLC27 mRNA expression in Oncomine database. The comparison indicates the number of datasets with higher (right column, red) and lower (left column, blue) SLC27 mRNA expression when compared to normal tissue; (**b**) The box plot comparing specific SLC27A4 expression in normal (*n* = 61, labeled as (1) and breast cancer (*n* = 389, invasive ductal breast carcinoma cancer tissue, labeled as (2) was derived from the The Cancer Genome Atlas (TCGA) Breast dataset of Oncomine database; (**c**) The correlation between SLC27A4 RNA expression levels and overall survival time according RNA-sequencing data from Cancer Genome Atlas in Human Protein Atlas (https://www.proteinatlas.org) database; (**d**) The correlation between SLC27A4 RNA expression (probe: 225779_at) and distant metastasis free survival (DMFS) in Kaplan-Meier (KM)-plotter database (http://kmplot.com); (**e**) The SLC27A4 protein expression in normal breast and breast cancer tissues was analyzed through the Human Protein Atlas database. Scale bar = 200 mm; (**f**) The staining intensity of SLC27A4 in 12 breast cancer tissues in Human Protein Atlas database. The SLC27A4 expression was further evaluated by the UALCAN database according to (**g**) different subtypes; (**h**) different stages; and (**i**) different races in TCGA breast cancer samples. The number in parentheses indicates sample size in each group. In the box plots, the boundary of the box respectively indicates the lower and upper quantile and the black line within the box indicates the median. * *p* < 0.05, ** *p* < 0.01, *** *p* < 0.001 as compared between each group.

The expression levels of SLC27 mRNA in normal and cancer tissues were evaluated in the Oncomine database. Datasets selection was based on the threshold, *p*-value of 0.001, fold change of 2, and being in the top 10% gene ranking. In Figure 1a,b, the results show that only SLC27A4 expression was higher in breast cancer tissues than that in nontumor tissues. The other SLC27 family proteins showed opposite expression pattern. The data implies that SLC27A4 might be associated with malignancy of breast cancer. In addition, there is a trend toward shorter overall survival and distant metastasis-free survival in breast cancer patients with higher SLC27A4 expression (Figure 1c,d, *p* = 0.0725 and 0.033 respectively). By contrast, the high expression SLC27A1 and SLC27A6 was associated better overall survival rate (Supplementary Figure S1). The SLC27A4 protein expression in normal breast and breast cancer tissues were also evaluated by the Human Protein Atlas database (Figure 1e). Compared to normal breast tissues, most breast cancer tissues revealed median to high SLC27A4 expression (Figure 1f). To further investigate whether SLC27A4 expression was associated with different subtypes of breast cancer, different stages, and races in clinical patients, the UALCAN database was used. Our results showed that significantly higher SLC27A4 expression was observed in all subtypes, stages, and races in breast cancer tissues when compared to normal breast tissue (Figure 1g–i). No significantly different levels of SLC27A4 were shown among all cancer stages; however, significant differences between luminal vs. triple negative (*p* < 0.0001) and HER2 positive vs. triple negative (*p =* 0.0180) in different subtype analysis, and Caucasian vs. African American (*p =* 0.0013) and Caucasian vs. Asian (*p =* 0.0174) in different race analysis were observed. In general, SLC27A4 mRNA expression in breast tumor tissues was higher than that in normal breast tissues in clinical samples.

### 2.2. Silencing SLC27A4 in Breast Cancer Cell LINES Results in Decreasing Fatty Acids Uptake Capacity

**Figure 2:**
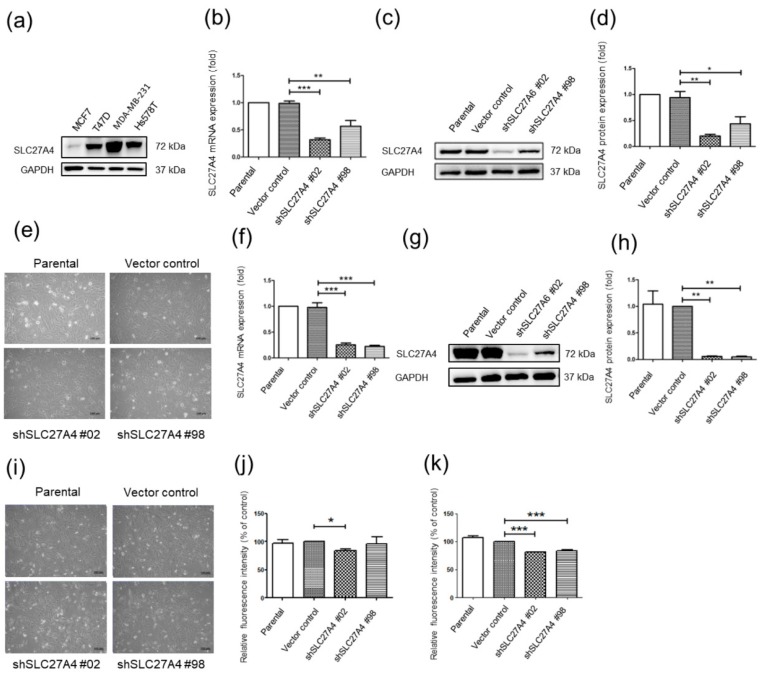
Knockdown SLC27A4 gene expression in breast cancer cell lines. (**a**) SLC27A4 protein expression in breast cancer cell lines. In SLC27A4 shRNA-transfected Hs578T; (**b**) SLC27A4 mRNA expression; (**c**) SLC27A4 protein expression; (**d**) quantification of protein expression (*n* = 3); and (**e**) cell morphology were shown. In SLC27A4 shRNA-transfected MDA-MB-231; (**f**) SLC27A4 mRNA expression; (**g**) SLC27A4 protein expression; (**h**) quantification of protein expression (*n* = 3); and (**i**) cell morphology were shown. Fatty acid uptake assay in (**j**) Hs578T (*n* = 3) and (**k**) MDA-MB-231 (*n* = 3). * *p* < 0.05, ** *p* < 0.01, *** *p* < 0.001 as compared with the vector control. In the bar plots, the mean ± standard error of mean (SEM) was shown. * *p* < 0.05, ** *p* < 0.01, *** *p* < 0.001 as compared between each group. Scare bar = 100 μm.

The SLC27A4 expression was evaluated by Western blot assay in luminal A breast cancer cell lines T47D and MCF-7, and triple negative breast cell lines Hs578T and MDA-MB-231 (Figure 2a) [15]. Except for MCF7, the other three cell lines express high levels of SLC27A4 protein. Hs578T and MDA-MB-231 were chosen for the following experiments. Two different targeted sequences of short hairpin RNA (shRNA), shSLC27A4#98 and shSLC27A4#02, were used for silencing SLC27A4 expression in Hs578T and MDA-MB-231. Because inhibition of fatty acid synthase mediates epithelial-mesenchymal transition (EMT) in the breast through FABP1 and other proteins [16], the cell morphology of SLC27A4-silencing cells was also evaluated. Figure 2b–d reveal that shSLC27A4#98 and shSLC27A4#02 effectively suppressed SLC27A4 in Hs578T, and Figure 2e shows the morphology of shSLC27A4-knockdowned Hs578T. Furthermore, the effect of shSLC27A4#98 and shSLC27A4#02 in MDA-MB-231 is shown in Figure 2g–i. Because the enzyme function of SLC27 family links to fatty acids transport [12], the fatty acids uptake capacity was evaluated in both cell lines. In Hs578T, only the shSLC27A4#02-transfected group revealed lower fatty acids uptake capacity when compared to the vector control group (Figure 2j). By contrast, relatively low fatty acids uptake was detected in shSLC27A4#98- and shSLC27A4#02-transfected MDA-MB-231 (Figure 2k). Our results suggest that the fatty acids uptake capacity was associated with silencing efficiency in two breast cancer cell lines.

### 2.3. Silencing SLC27A4 in Breast Cancer Cell Lines Inhibited Cell Growth

**Figure 3:**
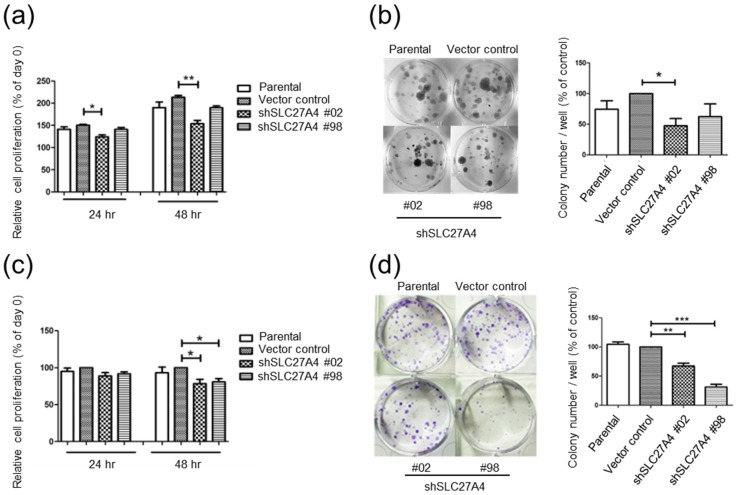
Silencing SLC27A4 gene expression inhibited cell growth in breast cancer cell lines. Cell proliferation in Hs578T was evaluated by (**a**) WST-1 assay (*n* = 3) and (**b**) colony formation assay (*n* = 3). Cell proliferation in MDA-MB-231 was evaluated by (**c**) WST-1 assay (*n* = 4) and (**d**) colony formation assay (*n* = 3). * *p* < 0.05, ** *p* < 0.01, *** *p* < 0.001, as compared with the vector control. The mean ± SEM was shown in bar plots.

The cell proliferation in SLC27A4-knockdowned Hs578T and MDA-MB-231 was evaluated. In Hs578T, cell growth was inhibited after transfection with shSLC27A4#02 but not shSLC27A4#98 when compared to the vector control group (Figure 3a,b). In MDA-MB-231, relatively slower cells growth rate was observed in both shSLC27A4#02 and shSLC27A4#98-transfected groups (Figure 3c,d). It is interesting to note that the inhibitory effect on cell growth in both cells was associated with the inhibitory efficiency of SLC27A4 shRNAs (Figure 1c,g). This suggested that SLC27A4 is associated with enhancement of cell growth in breast cancer cells.

### 2.4. Silencing SLC27A4 in Breast Cancer Cell Lines Affected G0/G1 and G2/M Cell Cycle

**Figure 4:**
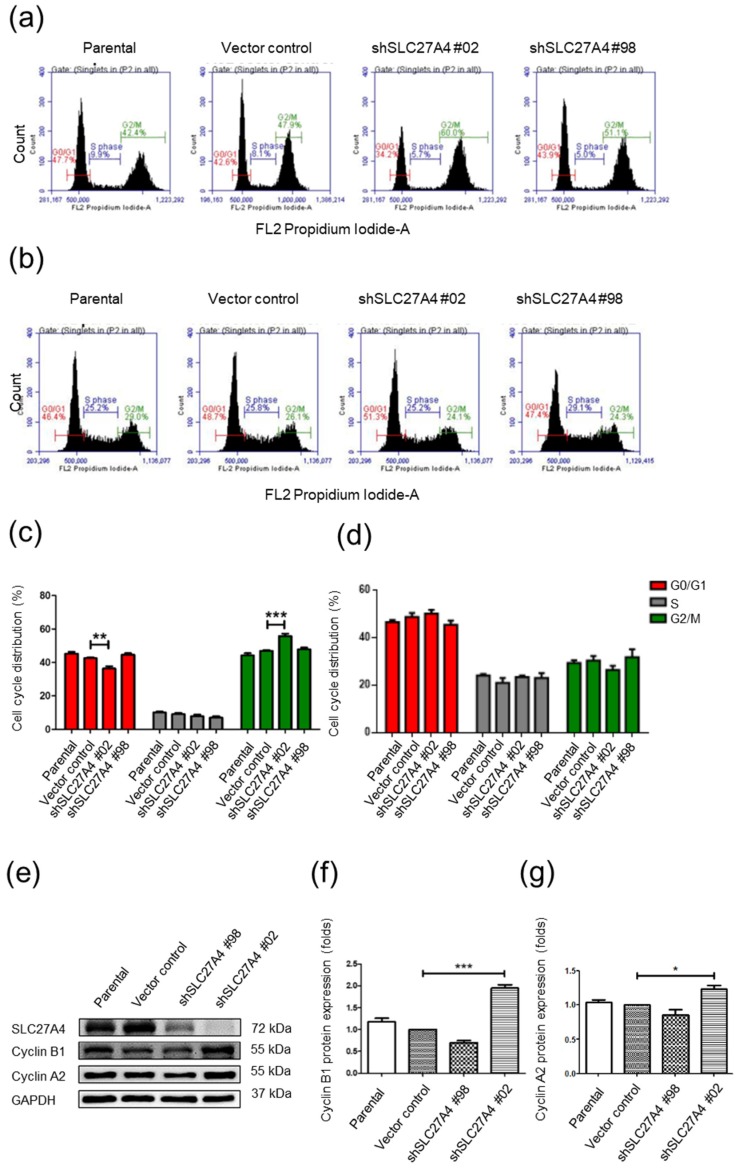
Silencing SLC27A4 gene expression affected cell cycle in Hs578T. Cell cycle analysis was performed via propidium iodide (PI) staining. (**a**) Flow cytometry analysis of SLC27A4 silencing Hs578T cells and (**b**) SLC27A4 silencing MDA-MB-231 cells. The quantitative results of cell cycle analysis (**c**) Hs578T (*n* = 4) and (**d**) MDA-MB-231 (*n* = 4). (**e**) In Hs578T, the protein expression of cell cycle regulators cyclin B1 and cyclin A2, and the quantitative result of Western blot assay: (**f**) cyclin B1 (*n* = 3) and (**g**) cyclin A2 (*n* = 6). * *p* < 0.05, ** *p* < 0.01, *** *p* < 0.001 as compared with the vector control. The mean ± SEM was shown in bar plots.

The cell cycle status in SLC27A4-silencing Hs578T and MDA-MB-231 was analyzed. The propidium iodide staining assay showed that decreasing cell population in G0/G1 phase and increasing cell population in G2/M phase in the shSLC27A4#02 group but not the shSLC27A4#98 group of Hs578T (Figure 4a). However, SLC27A4 silencing did not significantly affect the cell cycle in MDA-MB-231 (Figure 4b). The quantitative results are shown in Figure 4c,d. In addition, the regulator proteins of cell cycles were investigated in Hs578T. Figure 4e–g show that the protein expression levels of cyclin B1 and cyclin A2 significantly increased in the shSLC27A4#02 group.

### 2.5. Silencing SLC27A4 in Breast Cancer Cell Lines Inhibited Capacity of Migration and Invasion

**Figure 5:**
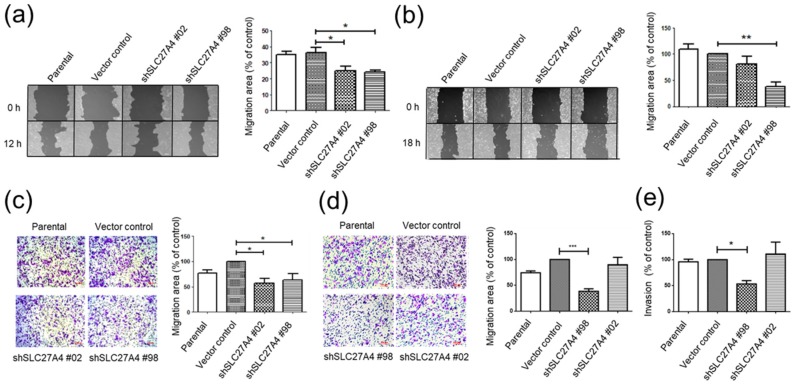
Silencing SLC27A4 gene expression inhibited cell migration and invasion in breast cancer cell lines. Cell migration capacity was evaluated by wound-healing assay in (**a**) Hs578T (*n* = 3) and (**b**) MDA-MB-231 (*n* = 3), and by transwell migration assay in (**c**) Hs578T (*n* = 3) and (**d**) MDA-MB-231 (*n* = 4). (**e**) Invasion capacity of SLC27A4-silencing MDA-MB-231 was shown (*n* = 4). * *p* < 0.05, ** *p* < 0.01, *** *p* < 0.001 as compared with the vector control. Scare bar = 100 μm. The mean ± SEM was shown in bar plots.

The cell migration in SLC27A4-knockdowned Hs578T and MDA-MB-231 was evaluated via wound-healing assay. The results revealed that cell migration capacity was significantly inhibited in both SLC27A4-silencing groups in Hs578T and in shSLC27A4#98 groups in MDA-MB-231 (Figure 5a,b). Furthermore, similar results were observed in the transwell migration assay (Figure 5c,d). Because MDA-MB-231 is a highly metastatic cell line, the invasion capacity was further determined. Our result indicated that the shSLC27A4#98 group had lower invasion capacity than that in the vector control group (Figure 5e). It suggests that the SLC27A4 expression level was also associated with migration and invasion capacity of breast cancer.

### 2.6. Silencing SLC27A4 in Breast Cancer Cell Lines Affected Regulatory Molecules of Epithelial Mesenchymal Transition Signaling Pathways

**Figure 6:**
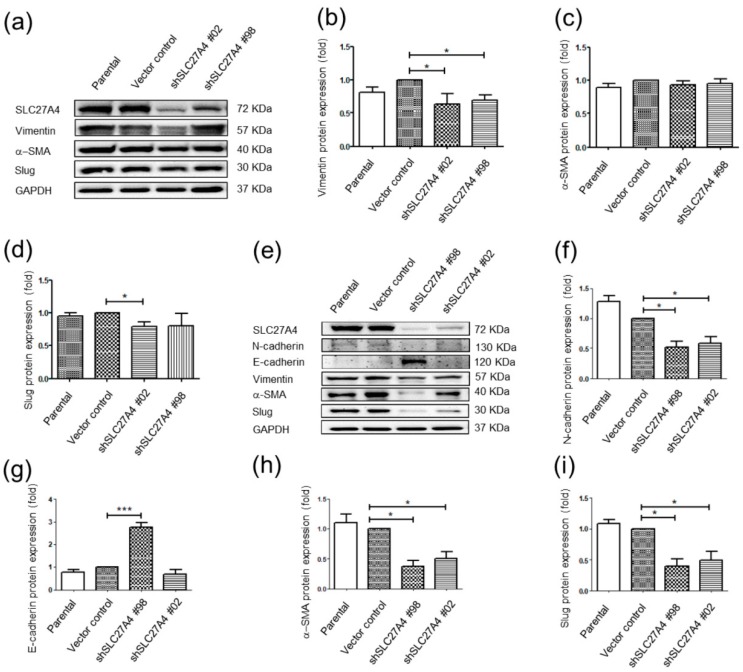
Silencing SLC27A4 gene expression affected regulatory molecules of EMT signaling pathways. (**a**) In Hs578T, the protein expression levels were determined by Western blot, and the quantitative result of (**b**) vimentin (*n* = 3), (**c**) α-SMA (*n* = 5), and (**d**) Slug (*n* = 5). (**e**) In MDA-MB-231, the protein expression levels were determined, and the quantitative result of (**f**) N-cadherin (*n* = 3), (**g**) E-cadherin (*n* = 5), (**h**) α-SMA (*n* = 3), and (**i**) Slug (*n* = 3). * *p* < 0.05, *** *p* < 0.001 as compared with the vector control. The mean ± SEM was shown in bar plots.

The migration and invasion capacity of Hs578T and MDA-MB-231 was inhibited after silencing SLC27A4. EMT phenotype is associated with metastasis and invasion in cancer cells [17]. Increasing N-cadherin, vimentin, Slug, and α-smooth muscle actin (α-SMA) and decreasing E-cadherin are biomarkers of EMT signaling [17]. Therefore, the regulatory molecules of EMT introduction was investigated. In Hs578T, the protein expression levels of vimentin and Slug decreased in SLC27A4 silencing groups. The expression of α-SMA was not significantly changed (Figure 6a–d). In MDA-MB-231, the expression of N-cadherin, E-cadherin, Slug, and α-SMA was significantly affected in SLC27A4 silencing groups; however, the expression of vimentin was not changed. These results suggest that knockdown of SLC27A4 affected the regulatory molecules of EMT signaling pathways.

### 2.7. Potential Interacting Networks in SLC27A4-Silencing Breast Cancer Cells

**Figure 7:**
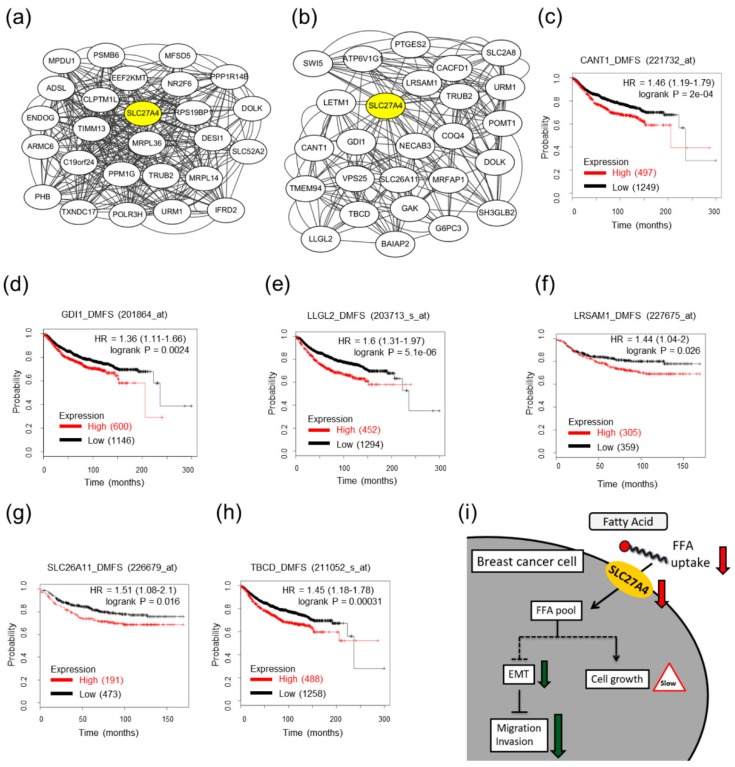
Potential interacting networks of SLC27A4. (**a**) The interacting networks of SLC27A4 in normal breast mammary tissues; (**b**) The interacting networks of SLC27A4 in breast cancer tissues. The top-25 SLC27A4-associated genes that have the highest score in TCSBN database are shown. The correlation between (**c**) CANT1; (**d**) GDI1; (**e**) LLGL2; (**f**) LRSAM1; (**g**) SLC27A11; and (**h**) TBCD mRNA expression and distant metastasis free survival (DMFS) in KM-plotter database; (**i**) The summary scheme of this study. Knockdown of SLC27A4 in breast cancer cells cause inhibition of cell growth, migration, and invasion. The dashed lines indicate the unknown regulatory mechanism, and the continuous lines indicate the conclusion which is supported by experimental evidences.

To the best of our knowledge, the detailed regulatory mechanism of SLC27A4 is still unknown in breast cancer. Therefore, the biological networks of SLC27A4 were evaluated by TCSBN database, which provided biological networks in various types of cancer and non-cancer tissues [18]. In this study, the interacting networks were drawn according to the top-25 genes with high co-expression correlation among normal and cancer tissues (Figure 7a,b). The results showed that most genes within interacting networks in breast cancer tissues were different from that in normal breast mammary tissues. Only three genes including *Dolichol Kinase* (*DOLK*), *TruB Pseudouridine Synthase Family Member 2* (*TRUB2*), and *Ubiquitin Related Modifier 1* (*URM1*) were identical between normal and cancer tissues. In order to determine whether the function of the these genes, these 25 genes in normal breast and breast cancer tissues were performed the functional annotation bioinformatics analysis through DAVID Bioinformatics Resources [19,20]. The results were shown in Supplementary Tables S1 and S2, respectively. The analysis showed that SLC27A4-correlated genes mainly involved in metabolic processes in normal breast tissues and in transport processes in breast cancer tissues. In top-25 SLC27A4 co-expression genes in breast cancer tissues, six genes including *Solute carrier family 26 member 11* (*SLC26A11*), *calcium activated nucleotidase 1* (*CANT1*), *leucine rich repeat and sterile alpha motif containing 1* (*LRSAM1*), *tubulin folding cofactor D* (*TBCD*), *LLGL2 scribble cell polarity complex component* (*LLGL2*), and *GDP dissociation inhibitor 1* (*GDI1*) were associated with poor DMFS (Figure 7c–h). These evidences might provide a possible interacting network of SLC27A4 in clinical breast cancer tissues. The summarized graph of the present study is presented in Figure 7i.

## 3. Discussion

The bioinformatic analysis revealed that high SLC27A4 was associated with breast cancer tissue and poor prognosis in breast cancer patients. In addition, our results suggest that silencing SLC27A4 expression inhibited cell growth, migration and invasion capacity in breast cancer cell lines. Bioinformatic analyses of SLC27A4-interacting network are linked to several types of metabolic pathways and regulation of cell size. Phospholipids are essential components for all membranes. During growth and cell cycle progression, the regulation of DNA synthesis and phospholipids synthesis/turnover must be integrated. The intracellular fatty acids pool is contributed from de novo fatty acid synthesis and extracellular fatty acid transport in breast cancer cells [21]. Therefore, we supposed that SLC27A4-silencing should affect cell growth and cell cycle in both cell lines. The present results showed that cell growth was inhibited after SLC27A4-silencing. G2/M cell cycle arrest was observed in in shSLC27A4#02 Hs578T and increased protein expression of cyclin A2 and cyclin B1 was detected. Cyclin A2 and cyclin B1 activated cyclin-dependent kinase 1 (CDK1), which regulates mitotic entry and progression [22]. Cyclin A2 regulates nuclear-envelope breakdown and then the cyclin B1-CDK1 complex is activated [23]. A previous study demonstrated that inhibition of fatty acid synthase activity arrested the cancer cells at G2/M [24]. When cancer cells are treated with the inhibitor of fatty acid synthase, the increased protein expression of cyclin B1 was observed. The cyclin A2 expression was significantly affected by the inhibitor [24].

Fatty acid synthase and acetyl-CoA carboxylase 1 are critical enzymes involved in de novo fatty acid synthesis [25]. It suggests that interference of intracellular fatty acids pool arrests cells at late G2/mitosis before anaphase/telophase. The degradation pathways of cyclin A2 and cyclin B1 might be attenuated by SLC27A4-silencing, and subsequently contribute to G2/M arrest. Perhaps CDK1 is involved in this cell cycle regulation in Hs578T. Interestingly, the cell cycle of SLC27A4-silencing MDA-MB-231 was not significantly changed. Knockdown of acetyl-CoA carboxylase 1 or fatty acid synthase disrupts fatty acids synthesis, acetyl-CoA and CoA production, and then induces apoptosis in breast cancer cells [26,27]. Therefore, SLC27A4-silencing might also lead to slight cell death but not affect cell cycle progression. It will be further investigated in the future.

The cell cycle results were inconsistent between MDA-MB-231 and Hs578T. Hs578T is a cell line derived from primary tumor and its pathology is distinguishing infiltrating ductal carcinoma [28]. By contrast, MDA-MB-231 is derived from pleural effusion and its pathology is distinguishing adenocarcinoma [28]. Both cells have mutant p53, and wild type BRCA1. Hs578T has a *HRAS* mutation, and MDA-MB-231 has *B-Raf Proto-Oncogene* (*BRAF*), *Cyclin Dependent Kinase Inhibitor 2A* (*CDKN2A*, *p16*), and *KRAS* mutation [28,29]. In mammary cells, p53 is a key regulator of cell cycle [30,31]. Because Hs578T has a mutant p53, p53 should not play a role in SLC27A4-mediated cell cycle regulation in Hs578T. The other tumor suppressor, such as wild type p16, might be important for regulating cell cycle in SLC27A4-silencing Hs578T.

In this study, the capacity of migration and invasion was suppressed after silencing SLC27A4 (Figure 5). Generally, mesenchymal phenotype is usually associated with tumor migration, invasion, and poor clinical outcomes [32]. A previous study decreasing FASN and FABP1 cause inhibition of EMT in breast cancer cells [16]. The cell morphology of SLC27A4-silencing Hs578T and MDA-MB-231 was not significantly affected when compared to control groups. The Western blot analyses showed that the expression of transcription factors that promoted EMT was suppressed in SLC27A4-silencing cells. Moreover, bioinformatic analyses suggest that SLC27A4 is involved in regulation of cell size in breast cancer cells. Transforming growth factor-β (TGF-β) induced EMT and increased cell size through mammalian target of rapamycin (mTOR) signaling pathways [33]. This data might suggest interaction between SLC27A4-mediated cell size regulation and TGF-β signaling pathways. BRAF is involved in the processes of EMT, stemness or metastasis in breast [34]; thus, different BRAF status in two breast cancer cell lines might affect the EMT signaling pathways after SLC27A4 silencing.

Palmitic acid (a common saturated fatty acid, C16:0) or a high-fat diet enhances the metastatic potential of CD36^+^ metastasis-initiating breast cancer cells [11]. Interestingly, a previous study indicated that CD36 enhances fatty acid uptake but does not transport fatty acid across the plasma membrane in a mammalian cell line [35]. It implies that uptake of palmitic acid or other types of fatty acid is not fully dependent on CD36 in breast cancer. The other families of transporters are necessary for utilization of fatty acid outside cells. ACSL4 has a fatty acid transporter activity and its preferred substrate is arachidonic acid (a unsaturated fatty acid, C20:4) [36]. Silencing ACSL4 in breast cancer cells affects the components of cell membranes, especially arachidonic acid [37]. Arachidonic acid is also known as a fatty acid that links to cancer metastasis [38]; on the other hand, palmitic acid and lignoceric acid (C24:0) are known substrates of SLC27A4 [13]. In Figure 2j–k, low capacity of fatty acid uptake was detected in SLC27A4-silencing Hs578T and MDA-MB-231. We speculate that knockdown of SLC27A4 might alter uptake of specific fatty acids and then change the composition of intracellular fatty acids pool. When compared to the substrates among SLC27A4, SLC27A1 and SLC27A6, oleic acid (C18:1) is a preferred substrate of SLC27A1 and SLC27A6, but not SLC27A4 [13]. Oleic acid has revealed antitumor effects in several types of cancers [39]. Perhaps this is why SLC27A4 expression is opposite to other SLC27 family proteins expression. Because Hs578T and MDA-MB-231 were maintained at media with normal fetal bovine serum that contained various types of fatty acids in the present study, we could not evaluate the effect of each suspicious fatty acid in SLC27A4-silencing cells. Culture medium fatty-acid withdrawal via Bio-Beads methods might be a strategy to investigate whether fatty acids are important factors to regulate cell growth, migration, and invasion. These issues will be further investigated in the future.

Currently, the interaction of SLC27A4 is not well-known in cancer cells. In lung cancer cell lines, SLC27A4 directly interacts with autophagy-related 4B cysteine peptidase (ATG4B) [40]. There are no related studies indicating the interacting networks of SLC27A4 in breast cancer. Because modulation of a metabolic enzyme expression might affect the entire metabolic flux, investigating the interacting networks is essential for further studies. Thus, the TCSBN database was used for predicting the possible interacting networks and DAVID Bioinformatics Resources was used for understanding the functions of these genes. In high SLC27A4-expressing breast cancer tissues and low SLC27A4-expressing normal breast tissues, different biological processes were observed. In addition, our analysis revealed six SLC27A4-correlated genes including CANT1, GDI1, LLGL2, LRSAM1, SLC26A11, and TCBD were also associated poor prognosis in clinical samples. An emerging study demonstrates that LLGL2 involves in Hippo-YAP pathway which regulates bone metastasis in breast cancer [41]. The function of other genes has not been investigated in breast cancer. It is worth investigating the interactions among SLC27A4 and these genes in breast tumor cells and tumor environment in future studies. We believe that SLC27A4 is a potential diagnostic marker for breast cancer. Because SLC27A4 is a transmembrane protein, blockage of extracellular SLC27A4 via a SLC27A4 antibody might be a novel therapeutic strategy against breast cancer due to disruption of the SLC27A4/CD36-mediated fatty acids transportation pathway. This issue should be further evaluated in animal tumor models and clinical specimens in the future.

## 4. Materials and Methods

### 4.1. Cell Culture

Human mammary cancer cell lines MDA-MB-231 (HTB-26™), Hs578T (HTB-126™), T47D (HTB-133™), and MCF-7 (HTB-22™) were purchased from American Type Culture Collection (Manassas, VA, USA). MCF-7, Hs578T, and T47D was respectively cultured in Minimum Essential Medium (MEM), Dulbecco’s Modified Eagle Medium (DMEM), and RPMI1640 supplied with 10% fetal bovine serum (Life Technologies, Grand Island, NY, USA), 100 units/mL penicillin G, 100 μg/mL streptomycin, and 0.25 μg/mL amphotericin B in 5% CO_2_ air atmosphere at 37 °C. In addition, MDA-MB-231 was cultured in Leibovitz’s L-15 Medium with 10% FBS, 100 units/mL penicillin G, 100 μg/mL streptomycin, and 0.25 μg/mL amphotericin B in a CO_2_-free air atmosphere at 37 °C. All culture media and supplements were purchased from Lonza (Walkersville, MD, USA).

### 4.2. Bioinformatic Analysis

The expression in SLC27 gene family (SLC27A1-6) in breast cancer samples and nontumor breast samples across available datasets was evaluated by Oncomine Research Edition (http://www.oncomine.org, v4.5; Thermo Fisher Scientific, Inc., Waltham, MA, USA). The expression of SLC27A4 was further evaluated in the TCGA breast dataset in Oncomine Research Edition, either. Moreover, the expression of SLC27A4 in different subtypes and races of breast cancer samples was evaluated by the UALCAN (http://ualcan.path.uab.edu) [42]. The association between gene expression and overall survival rate of breast cancer patients was obtained from the Human Protein Atlas (https://www.proteinatlas.org) [43,44]. In addition, the images of SLC27A4 protein expression in normal breast and breast cancer tissues, and results staining intensity of SLC27A4 were obtained from the Human Protein Atlas database (Antibody: HPA007293). The high and low expression groups were separated by “best separation” on the website of the Human Protein Atlas. Distant metastasis-free survival (DMFS) was evaluated by Kaplan–Meier (KM) plotter (http://kmplot.com) [45] and high- and low-expression groups were divided according to the “auto select best cutoff” in the website. The interacting networks were determined according to the TCSBN database (http://inetmodels.com) [18]. “Maximum number of nodes” was set at 25 and “Edge Pruning Parameter (-log10 P)” was set at 3 in the TCSBN database. The interacting networks were drawn by Cytoscape version 3.6.1 [46]. Functional annotation (biological process) was determined by DAVID Bioinformatics Resources (https://david.ncifcrf.gov) [19,20].

### 4.3. Western Blot Assay

To collect protein, cells were cultured in a 6-cm dish for 48 h and then were lysed in radioimmunoprecipitation lysis buffer (Millipore, Billerica, MA, USA) with protease inhibitor cocktail (Millipore) at a 1000:1 ratio. Protein concentration was determined by Pierce BCA Protein Assay Kit (Thermo Fisher Scientific, Billerica, MA, USA), then separated on 10–15% sodium dodecyl sulfate polyacrylamide gel electrophoresis (SDS-PAGE) and transferred to Polyvinylidene difluoride (PVDF) membranes (Millipore). After 1 h blocking with 5% dried skimmed milk in tris-buffered saline with Tween-20 (TBST) buffer, the membrane was hybridized with the primary antibodies including anti-E-cadherin (1:1000, Cat. No. #610182), anti-N-cadherin (1:1000, Cat. No. #610921), anti-Vimentin (1:3000, Cat. No. #550513) were purchased from BD Transduction Laboratories™; anti-GAPDH (1:5000, Cat. No. #MAB374) was purchased from Millipore (USA); anti-α-SMA (1:1000, Cat. No. #A5228) was purchased from Sigma-Aldrich (St. Louis, MO, USA); anti-Slug (1:1000, Cat. No. #9585S), anti-cyclin A2 (1:1000, Cat. No. #4656) and anti-cyclin B1 (1:1000, Cat. No. #4135) were purchased from Cell Signaling Technology (Danvers, MA, USA); anti-SLC27A4 (1:2000, Cat. No. #ab199719) was purchased from Abcam (UK) at 4 °C overnight. After TBST washing three times, the membrane was then hybridized with anti-rabbit IgG or anti-mouse IgG HRP-linked antibody (Cell Signaling Technology, USA). The results were acquired on Alpha Innotech FluorChem FC2 imaging system (ProteinSimple; Bio-Techne, Minneapolis, MN, USA).

### 4.4. Knockdown of SLC27A4

Lentivirus shRNAs were purchased from RNAi Core Facility (Taipei, Taiwan). The lentivurus-shRNA clones included: Lenti-emptyT (clone ID, TRCN0000089107; a vector control); Lenti-shSLC27A4 #98 (clone ID, TRCN0000043398; targeting sequence: 5′-CTTCACAGATAAACTGTTCTA-3′); Lenti-shSLC27A4 #02 (clone ID, TRCN0000043402; targeting sequence: 5′-CCGGGTCTTCATCAAGACCAT-3′). To silencing the gene expression, the Hs578T and MDA-MB-231 cells lines were complete culture media containing 8 μg/mL polybrene (EMD Millipore, Billerica, MA, USA) in a 6-cm dish at 37 °C for 30 min. Lentiviruses for Hs578T and MDA-MB-231 were added for infection at multiplicity of infection (MOI) = 5 and MOI = 3, respectively. After 24 h of incubation, the culture medium was refreshed with fresh culture media, with 2 μg/mL puromycin (Sigma-Aldrich; Merck KGaA, Darmstadt, Germany), for 48 h. The infected cells were maintained in medium with 2 μg/mL puromycin, and subsequently used in assays.

### 4.5. Fatty Acid Uptake Assay

The capacity of fatty acid uptake was determined by using the Free Fatty Acid Uptake Assay Kit (Fluorometric) according to the manufacturer’s instructions (cat. no. ab176768; Abcam, Cambridge, UK). Before fatty acid uptake assay, 1 × 10^4^ Hs578T and MDA-MB-231 were seeded on a 96-well plate overnight. The cells were preincubated in serum-free media for 1 h after phosphate-buffered saline washing. Subsequently, cells were incubated in a fluorescent fatty acid mixture for 30 min. The results were evaluated by using a microplate fluorescence reader at 485/528 nm (FL × 800; BioTek Instruments Inc., Winooski, VT, USA). The fluorescence signals from wells containing assay mix without cells were used as the background and fluorescence quantification in vector control groups was set to 100% for relative quantification.

### 4.6. Real-Time Quantitative PCR

Total RNA was isolated via TRIzol reagent (Invitrogen, Carlsbad, CA, USA) and reverse transcription of cDNA was performed via the PrimeScript RT reagent kit (Clontech Laboratories, Inc., Kusatsu, Japan). The relative PCR levels were determined by Fast SYBR-Green Master Mix (Applied Biosystems, Foster City, CA, USA) with the specific primer targeting human Solute Carrier Family 27 Member 4 (SLC27A4), 5′-TCCTGTGGGCTTTTGGTTGT-3′ and 5′-TGGCACCCAACTCAACACAT-3′, and human Glyceraldehyde-3-phosphate dehydrogenase (GAPDH), 5′-GAGTCAACGGATTTGGT CGT-3′ and 5′-TTGATTTTGGAGGGATCTCG-3′, on a Real-Time PCR system (StepOnePlus Real-Time PCT system; Applied Biosystems, Foster City, CA, USA). The relative mRNA expression was normalized to the GAPDH expression and calculated using the 2^−ΔΔ*C*t^ method [47].

### 4.7. WST-1 Assay

The cell proliferation of Hs578T and MDA-MB-231 was evaluated by WST-1 (4-[3-(4-iodophenyl)-2-(4-nitrophenyl)-2H-5-tetrazolio]-1,3-benzene disulfonate) (Clontech, Mountain View, CA, USA). Briefly, 3 × 10^3^ cells were respectively seeded in 96-well plates overnight. The culture media were replaced with 100 μL mixture containing 95 μL of fresh culture media and 5 μL of WST-1 reagent. For 24- or 48-h incubation, the absorbance at 450 nm was determined on a microplate spectrophotometer (PowerWave X340; BioTek, Winooski, VT, USA).

### 4.8. Colony Formation Assay

To determine the long-term effect, 500 cells were seeded in a six-well plate. Cell culture media were replaced each 3 days until 14 days after seeding. Colonies were stained with crystal violet (0.4 g/L; Sigma-Aldrich, St. Louis, MO, USA) and the number of colonies was counted.

### 4.9. Cell Cycle Analysis

Hs578T and MDA-MB-231 cells were harvested at 48 h incubation after subculture. Harvested cells were fixed with 70% ethanol overnight at 4 °C and then were washed by phosphate-buffered saline. Subsequently, cells were incubated with 1 U/mL of DNase-free RNase A and 5 μg/mL of propidium iodide for 30 min at 4 °C in the dark (Sigma-Aldrich, St. Louis, MO, USA). The cell cycle distribution was determined on a flow cytometry (BD Accuri C6 flow cytometer, BD Biosciences San Jose, CA, USA). Amount of G0/G1, S and G2/M phase cells were determined as a percentage of the total number of cells.

### 4.10. Wound Healing Assay

1.5 × 10^5^ breast cancer cells were seeded into 24-well plates. A scratch was made by a 200 μL pipette tip when cells reached a complete confluent monolayer. After scratching, the suspended debris was removed by phosphate-buffered saline (PBS) washing. Subsequently, the cells were cultured in serum-free culture media (MDA-MB-231) or culture media with 1% FBS (Hs578T) for 24 h. The images were captured via a Leica inverted microscope and quantification was performed by TScratch software (version 1.0. Available at http://www.cse-lab.ethz.ch).

### 4.11. Transwell Migration and Invasion Assay

Before performing the transwell migration assay, 3 × 10^4^ breast cancer cells were seeded into a 24-well insert (Millicell Cell Culture Inserts 24-well Hanging Inserts, 8-μm PET, Millipore, St. Charles, MO, USA) in 300-μL serum-free medium, while 500 μL medium with 10% FBS was placed in the lower chamber. After culturing for 24 h, the transwell membrane on the 24-well insert was fixed with 500 μL 4% formaldehyde solution followed by 1% crystal violet staining. After removal of the cells on the upper surface, four images of each bottom membrane were captured using a Leica inverted microscope at ×100 magnification via Leica Applications Suite version 4.5.0™ (LAS v4.5) software (Leica Microsystems, Wetzlar, Switzerland). Invasion assay was performed by QCM ECMatrix Cell Invasion Assay, 24-well (8 μm), fluorimetric (Millipore, Billerica, MA, USA) according to the manufacturer’s instruction. Briefly, 1.2 × 10^5^ cells in 300 μL serum-free culture media was added in the insert and 500 μL media with 10% FBS was placed in the lower chamber for 48 h. The results were evaluated on a Bio-tek FLX-800 Fluorescence & Luminescence Reader at the excitation (Ex) and emission (Em) wavelengths = 485/528 nm.

### 4.12. Statistics

All graphs and statistics were made by the GraphPad Prism 7 software (GraphPad Software, Inc., La Jolla, CA, USA). To examine statistical differences among all groups, a one-way analysis of variance (ANOVA) with Tukey’s multiple comparison test was used. *p* < 0.05 was considered to indicate a statistically significant difference.

## 5. Conclusions

High expression of SLC27A4 was associated with breast cancer tissues and poor prognosis in breast cancer patients. In addition, knockdown of SLC27A4 decreased not only fatty acid uptake capacity in Hs578T and MDA-MB-231 but cell growth in Hs578T also, as well as capacity of migration and invasion in Hs578T and MDA-MB-231. Although detailed regulatory signaling pathways of SLC27A4 was not completely investigated in this study, our results firstly demonstrated that SLC27A4 was involved in progression of breast cancer. It is worth investigating whether SLC27A4 could serve as a diagnostic marker and treatment target in further studies.

## Supplementary Materials

Supplementary materials can be found at http://www.mdpi.com/1422-0067/19/11/3434/s1.
