# Supplementary material for: Solute Carrier Family 27 Member 4 (SLC27A4) Enhances Cell Growth, Migration, and Invasion in Breast Cancer Cells
Source: Int J Mol Sci. 2018 Nov 1;19(11):3434. doi: 10.3390/ijms19113434 (PMC6274775; doi:10.3390/ijms19113434)
Supplement: Supplementary file 1 [file ijms-19-03434-s001.pdf]

Solute Carrier Family 27 Member 4 (SLC27A4) enhances cell growth, migration, and invasion in breast cancer cells

Meng-Chi Yen, Shih-Kai Chou, Jung-Yu Kan, Po-Lin Kuo, Ming-Feng Hou and Ya-Ling Hsu

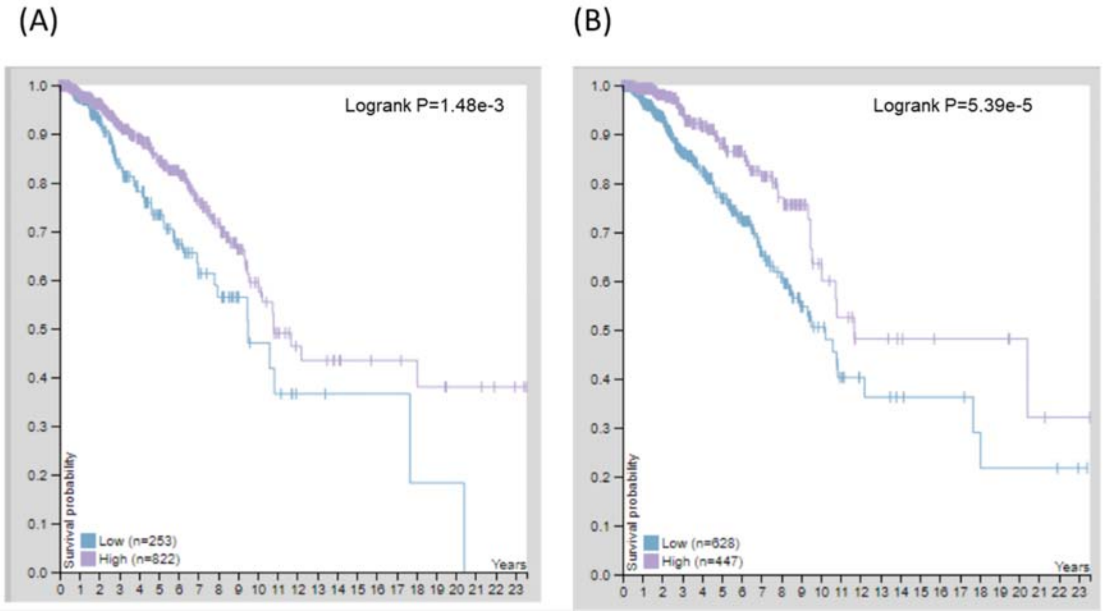

Figure S1. The correlation between (A) SLC27A1 and (B) SLC27A6 RNA expression levels and overall survival time according RNA-sequencing data from Cancer Genome Atlas in Human Protein Atlas database.

Table S1. The gene list of biological processes analysis in normal breast tissues

**Gene ontology enrichment-biological process**	**Genes symbols[1]**
Cellular metabolic process / primary metabolic process	*PPM1G, URM1, POLR3H, PSMB6, MRPL14, ENDOG, PHB, MPDU1, MRPL36, ADSL, TRUB2, DOLK, SLC27A4*
Dolichyl monophosphate biosynthetic process	*DOLK*
Protein urmylation / tRNA thio-modification / tRNA wobble uridine modification / tRNA wobble base modification	*URM1*
RNA modification	*URM1, TRUB2*
Cellular lipid metabolic process	*MPDU1, DOLK, SLC27A4*

[1] Gene symbols were shown in the "Gene" column. The genes that are involved in each biological process are listed in this Table. The other genes which are not significantly enriched in a specific biological process ($p > 0.05$) are not shown.

Table S2. The gene list of biological processes analysis in breast cancer tissues

Gene ontology enrichment-biological process	Genes symbols[1]
Transport / establishment of localization / localization	*SLC2A8, GDI1, LRSAM1, PTGES2, ATP6V1G1, NECAB3, SLC26A11, G6PC3, LLGL2, VPS25, SLC27A4*
Glucose transport / hexose transport / monosaccharide transport	*SLC2A8, G6PC3*
Cellular ketone metabolic process	*COQ4, PTGES2, G6PC3, SLC27A4*

[1] Gene symbols were shown in the "Gene" column. The genes that are involved in each biological process are listed in this Table. The other genes which are not significantly enriched in a specific biological process ($p > 0.05$) are not shown.
